# Supplementary material for: Time-series transcriptome analysis identified differentially expressed genes in broiler chicken infected with mixed Eimeria species
Source: Front Genet. 2022 Aug 8;13:886781. doi: 10.3389/fgene.2022.886781 (PMC9393255; doi:10.3389/fgene.2022.886781)
Supplement: Supplementary file 2 [file DataSheet1.ZIP › 4dpi_KEGG.Gsea.1625100298584/KEGG_ADHERENS_JUNCTION.html]

Details for gene set KEGG\_ADHERENS\_JUNCTION[GSEA]

|  || Dataset | TMM\_4dpi\_gct\_format\_4dpi\_gct\_format.Class\_4dpi.cls #PC\_versus\_NC.Class\_4dpi.cls #PC\_versus\_NC\_repos |
| Phenotype | Class\_4dpi.cls#PC\_versus\_NC\_repos |
| Upregulated in class | 1 |
| GeneSet | KEGG\_ADHERENS\_JUNCTION |
| Enrichment Score (ES) | 0.39917305 |
| Normalized Enrichment Score (NES) | 1.4488192 |
| Nominal p-value | 0.035081968 |
| FDR q-value | 0.3462812 |
| FWER p-Value | 0.9962 |
Table: GSEA Results Summary

  

Fig 1: Enrichment plot: KEGG\_ADHERENS\_JUNCTION      
 Profile of the Running ES Score & Positions of GeneSet Members on the Rank Ordered List

  

| SYMBOL | TITLE | RANK IN GENE LIST | RANK METRIC SCORE | RUNNING ES | CORE ENRICHMENT || 1 | LMO7 | na | 402 | 1.060 | 0.0164 | Yes |
| 2 | TCF7L2 | na | 607 | 0.874 | 0.0406 | Yes |
| 3 | ACTN4 | na | 673 | 0.838 | 0.0746 | Yes |
| 4 | CTNND1 | na | 787 | 0.765 | 0.1013 | Yes |
| 5 | PTPRJ | na | 827 | 0.749 | 0.1333 | Yes |
| 6 | SMAD3 | na | 934 | 0.698 | 0.1574 | Yes |
| 7 | ACTB | na | 1026 | 0.663 | 0.1810 | Yes |
| 8 | SSX2IP | na | 1248 | 0.589 | 0.1903 | Yes |
| 9 | SNAI2 | na | 1276 | 0.583 | 0.2155 | Yes |
| 10 | SORBS1 | na | 1415 | 0.543 | 0.2296 | Yes |
| 11 | YES1 | na | 1460 | 0.530 | 0.2509 | Yes |
| 12 | BAIAP2 | na | 1602 | 0.495 | 0.2624 | Yes |
| 13 | MET | na | 1612 | 0.493 | 0.2849 | Yes |
| 14 | PTPRB | na | 1654 | 0.484 | 0.3043 | Yes |
| 15 | AFDN | na | 1708 | 0.473 | 0.3222 | Yes |
| 16 | RAC1 | na | 1737 | 0.468 | 0.3419 | Yes |
| 17 | CTNNA2 | na | 1774 | 0.461 | 0.3607 | Yes |
| 18 | WASF1 | na | 1949 | 0.431 | 0.3665 | Yes |
| 19 | ACP1 | na | 2015 | 0.420 | 0.3808 | Yes |
| 20 | CTNNA1 | na | 2131 | 0.400 | 0.3901 | Yes |
| 21 | RHOA | na | 2375 | 0.366 | 0.3870 | Yes |
| 22 | FER | na | 2602 | 0.335 | 0.3839 | Yes |
| 23 | VCL | na | 2609 | 0.335 | 0.3992 | Yes |
| 24 | PARD3 | na | 3629 | 0.205 | 0.3235 | No |
| 25 | ACTN1 | na | 3764 | 0.191 | 0.3214 | No |
| 26 | CTNNB1 | na | 3857 | 0.182 | 0.3222 | No |
| 27 | WASL | na | 3867 | 0.181 | 0.3300 | No |
| 28 | CDH1 | na | 4042 | 0.164 | 0.3232 | No |
| 29 | PTPRM | na | 4220 | 0.149 | 0.3154 | No |
| 30 | WASF2 | na | 4299 | 0.142 | 0.3156 | No |
| 31 | PTPN1 | na | 4467 | 0.127 | 0.3076 | No |
| 32 | SRC | na | 4500 | 0.124 | 0.3107 | No |
| 33 | MAPK3 | na | 4570 | 0.119 | 0.3106 | No |
| 34 | NLK | na | 4762 | 0.101 | 0.2993 | No |
| 35 | EGFR | na | 4861 | 0.092 | 0.2955 | No |
| 36 | FARP2 | na | 5072 | 0.073 | 0.2813 | No |
| 37 | CDC42 | na | 5190 | 0.061 | 0.2744 | No |
| 38 | TGFBR2 | na | 5639 | 0.022 | 0.2380 | No |
| 39 | CSNK2B | na | 5872 | 0.004 | 0.2187 | No |
| 40 | NECTIN1 | na | 5879 | 0.004 | 0.2184 | No |
| 41 | TCF7L1 | na | 5944 | -0.002 | 0.2131 | No |
| 42 | EP300 | na | 6138 | -0.018 | 0.1978 | No |
| 43 | TJP1 | na | 6261 | -0.027 | 0.1889 | No |
| 44 | MAP3K7 | na | 6443 | -0.042 | 0.1757 | No |
| 45 | CREBBP | na | 6837 | -0.074 | 0.1463 | No |
| 46 | FGFR1 | na | 6964 | -0.086 | 0.1398 | No |
| 47 | ERBB2 | na | 7090 | -0.097 | 0.1339 | No |
| 48 | CSNK2A1 | na | 7174 | -0.106 | 0.1320 | No |
| 49 | SNAI1 | na | 7275 | -0.116 | 0.1291 | No |
| 50 | NECTIN3 | na | 7357 | -0.123 | 0.1281 | No |
| 51 | WASF3 | na | 7507 | -0.136 | 0.1220 | No |
| 52 | FYN | na | 7681 | -0.151 | 0.1147 | No |
| 53 | IGF1R | na | 7943 | -0.173 | 0.1010 | No |
| 54 | CSNK2A2 | na | 8097 | -0.188 | 0.0971 | No |
| 55 | RAC3 | na | 9492 | -0.347 | -0.0032 | No |
| 56 | PTPN6 | na | 9540 | -0.354 | 0.0095 | No |
| 57 | TGFBR1 | na | 9592 | -0.361 | 0.0223 | No |
| 58 | TCF7 | na | 10215 | -0.447 | -0.0087 | No |
| 59 | INSR | na | 10568 | -0.511 | -0.0140 | No |
| 60 | LEF1 | na | 10632 | -0.526 | 0.0055 | No |
| 61 | RAC2 | na | 10712 | -0.541 | 0.0244 | No |
| 62 | PTPRF | na | 10715 | -0.541 | 0.0498 | No |
| 63 | CTNNA3 | na | 11880 | -1.240 | 0.0109 | No |
Table: GSEA details [plain text format]

  

Fig 2: KEGG\_ADHERENS\_JUNCTION      
 Blue-Pink O' Gram in the Space of the Analyzed GeneSet

  

Fig 3: KEGG\_ADHERENS\_JUNCTION: Random ES distribution      
 Gene set null distribution of ES for **KEGG\_ADHERENS\_JUNCTION**
